# Editorial: Physiological, Pathological Roles and Pharmacology of Insulin Regulated Aminopeptidase

**DOI:** 10.3389/fmolb.2021.685101

**Published:** 2021-04-23

**Authors:** Siew Yeen Chai, Hugo Gutiérrez-de-Terán, Efstratios Stratikos

**Affiliations:** ^1^Department of Physiology, Monash Biomedicine Discovery Institute, Monash University, Clayton, VIC, Australia; ^2^Department of Cell and Molecular Biology, Uppsala University, Uppsala, Sweden; ^3^Biochemistry Laboratory, Department of Chemistry, National and Kapodistrian University of Athens, Athens, Greece

**Keywords:** aminopeptidase, metabolism, trafficking, immune system, peptide hormones, central nervous system, glucose, fibrosis

Insulin-Regulated Aminopeptidase (IRAP) is a transmembrane zinc metalloprotease with several reported biological functions. IRAP belongs to the M1 family of aminopeptidases (EC 3.4.11.3) and is also known as cystinyl aminopeptidase, placental leucine aminopeptidase (PLAP), and oxytocinase. The reported biological functions of IRAP include: (i) the regulation of trafficking of glucose transporter 4 (Keller, 2003), (ii) the generation of antigenic peptides for cross-presentation (Saveanu et al., 2009), (iii) T-cell receptor signaling (Evnouchidou et al., 2020), (iv) the regulation of placental oxytocin levels (Tsujimoto et al., 1992), (v) not well-understood roles in cognition and other central nervous system functions possibly through the regulation of oxytocin and vasopressin levels in the brain (Herbst et al., 1997; Albiston et al., 2011; Bernstein et al., 2017) or altered glucose uptake (Fernando et al., 2008; Albiston et al., 2011; Ismail et al., 2017), and (vi) the regulation of organ fibrosis (T. Gaspari, personal communication). All these roles have been associated with at least one of the two functional components of IRAP: an extracellular *C-terminal* domain that contains the M1 exopeptidase catalytic site and a 110 amino-acid long cytosolic *N-terminal* domain, connected by a single transmembrane-spanning region. The *C-terminal* domain underlies the ability of IRAP to trim antigenic peptides and peptide hormones, whereas the *N-terminal* domain appears to control intracellular trafficking and signaling events. The structure of the extracellular domain has been recently solved and resembles several other enzymes of the M1 family of aminopeptidases, featuring a large internal cavity adjacent to the catalytic center, which can accommodate peptide substrates (Mpakali et al., 2015) (Figure 1). The *C-terminal* domain can dimerize and change conformations upon ligand binding (Mpakali et al., 2017). Very little is currently known about the structure and molecular interactions of the *N-termina*l domain.

**Figure 1:**
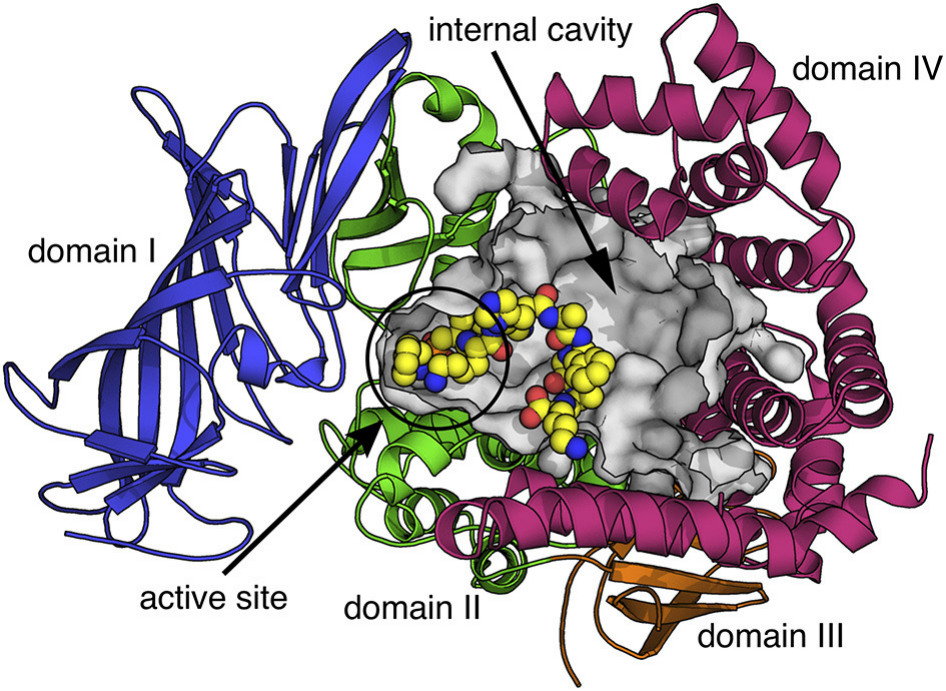
Schematic representation of the extracellular domain of IRAP based on the crystal structure with PDB code 4Z7I (Mpakali et al., 2015). Protein is depicted in cartoon representation with the four domains indicated in different colors. The internal cavity that contains the aminopeptidase active site and can accommodate peptide substrates is shown as a gray surface. A peptide analog co-crystallized with IRAP is shown in sphere representation (carbon = yellow, nitrogen = blue, oxygen = red, phosphorus = orange).

The important biological functions in which IRAP participates are attracting increased attention for possible pharmacological interventions. The primary function targeted to date has been the aminopeptidase activity, for which both functional and structural knowledge exists. In particular, IRAP inhibitors have been pursued as potential therapeutics for cognitive disorders (Chai et al., 2008; Andersson and Hallberg, 2012; Diwakarla et al., 2016), immune modulators (Kokkala et al., 2016), and more recently as anti-fibrotic agents.

In this special issue “Physiological, Pathological Roles and Pharmacology of Insulin Regulated Aminopeptidase,” we present a series of reviews and research papers written from leading authors in the field of IRAP, covering most aspects of the state-of-the-art research for this enzyme. With regards to the role of IRAP in metabolism, Trocmé et al. explore the possibility of using serum IRAP as a novel biomarker of prediabetes and type 2 diabetes, Krskova et al. demonstrate that IRAP inhibition improves glucose clearance in obese Zucker rats and Segarra et al. demonstrate how different types of dietary fat intake can affect IRAP and Alanyl aminopeptidase activities in the frontal cortex of the brain, the liver, and plasma. Exploring the role of IRAP in the immune system, Weimershaus et al. demonstrate how IRAP endosomes control phagosomal maturation in dendritic cells and Descamps et al. explore the role of IRAP in endocytic trafficking and receptor signaling in immune cells. Related to the role of IRAP in the central nervous system, Goto et al. describe a reciprocal relationship between IRAP expression and vasopressin levels in the murine brain. In view of the two functional domains of IRAP, Vear et al. explore the under-studied relationship between the *N-terminal* cytosolic and the *C-terminal* catalytic domains of IRAP and how the localization of IRAP may play an important role in defining its physiological or pathological functions. Finally, a number of articles explore the development of IRAP inhibitors with potential pharmacological and chemical biology applications: Georgiadis et al. provide a broad review on the development of IRAP inhibitors, Barlow and Thompson focus on efforts to develop inhibitors inspired by other members of the M1 family of aminopeptidases, Hallberg et al. review the development of angiotensin IV inspired small peptidemimetic inhibitors for IRAP and Vanga et al. explore the structural basis of inhibition of IRAP by benzopyran-based compounds.

It is becoming clear that the important biological functions played by IRAP will undoubtably sustain scientific interest on this enzyme in the coming years, especially in view of newly discovered functions with poorly understood molecular mechanisms. However, its multitude of biological roles could also act as a deterrent for drug development efforts. Likely, IRAPs tractability as a pharmaceutical target will greatly depend on detailed mechanistical analysis and prioritization of its biological functions, an aspect in which this Special Issue contributes. On the other hand, solid pre-existing progress in inhibitor design and development could incentivize drug development efforts as highlighted by contributed articles to this Special Issue. The possible effect of active-site inhibitors to IRAP's trafficking functions needs to be carefully addressed, an aspect that will certainly benefit from further work on the interplay of the two domains of IRAP. Regardless of these potential caveats, one thing is clear: the coming together of IRAP researchers from different fields and the continued frank and honest discussion that ensues will ensure that progress will be made, paving the way for the validation of IRAP as a tractable therapeutic target in the future.

## Author Contributions

All authors contributed to the writing of the editorial and have approved the final version.

## Conflict of Interest

The authors declare that the research was conducted in the absence of any commercial or financial relationships that could be construed as a potential conflict of interest.
